# Reoperations after first lumbar disc herniation surgery; a special interest on residives during a 5-year follow-up

**DOI:** 10.1186/1471-2474-8-2

**Published:** 2007-01-09

**Authors:** Arja Häkkinen, Ilkka Kiviranta, Marko H Neva, Hannu Kautiainen, Jari Ylinen

**Affiliations:** 1 Department of Physical Medicine and Rehabilitation, Jyväskylä Central Hospital, and Department of Health Sciences, University of Jyväskylä, Jyväskylä, Finland; 2 Department of Orthopaedics and Traumatology, Jyväskylä Central Hospital, Jyväskylä, Finland; 3 Department of Orthopaedic and Trauma Surgery, Tampere University Hospital, Tampere, Finland; 4 Rheumatism Foundation Hospital, Heinola, Finland; 5 Department of Physical Medicine and Rehabilitation, Jyväskylä Central Hospital, Jyväskylä, Finland

## Abstract

**Background:**

The overall rate of operations after recurrent lumbar disc herniation has been shown to be 3–11%. However, little is known about the rate of residives. Thus the aim of this study was to explore the cumulative rates of re-operations and especially residive disc herniations at the same side and level as the primary disc herniation after first lumbar disc herniation surgery and the factors that influence the risk of re-operations over a five year follow-up study.

**Methods:**

166 virgin lumbar disc herniation patients (mean age 42 years, 57% males) were studied. Data on patients' initial disc operations and type and timing of re-operations during the follow-up were collected from patient files. Back and leg pain on visual analog scale and employment status were collected by questionnaires.

**Results:**

The cumulative rate of re-operations for lumbar disc herniation was 10.2% (95% Cl 6.0 to 15.1). The rate of residives at initial site was 7.4% (95% Cl 3.7 to 11.3) and rate of lumbar disc herniations at other sites was 3.1% (95% Cl 0.6 to 6.2). The occurrence of residive lumbar disc herniations was evenly distributed across the 5 years. Neither age, gender, preoperative symptoms, physical activity nor employment had effect on the probability of re-operation.

**Conclusion:**

Seven percent of the lumbar disc patients had a residive lumbar disc operation within five years of their first operation. No specific factors influencing the risk for re-operation were found.

## Background

Surgery for lumbar disc herniation is effective in the majority of cases. Success rates from 76% to 93 % have been reported [1-8]. Patients who have had one operation for lumbar disc herniation have shown to be at 5–12.5% risk for further operations (including discectomy, other type of decompression or fusion) over the follow-ups lasting from 1 to 20 years [4,7,9,10]. The overall rate of operations after recurrent lumbar disc herniation has been shown to be 3–11% [11-14]. However, little is known about the rate of residives at the side and level with the primary operation.

In the current five-year follow-up study we analysed the cumulative rate of re-operations for lumbar disc surgery and especially rate of residive disc herniations after a first lumbar disc operation. The risk factors for repeated surgery were also studied.

## Methods

Two-hundred and ten patients had surgery for lumbar disc herniation in Jyväskylä Central Hospital in the year 1999 (~1/1000 inhabitant of the area). Of this number 173 patients (82%) volunteered for a follow-up study, filled a preoperative questionnaire and were referred for 2 and 12 month post-operative check-up visits in the hospital's out-patient clinic. Twelve-month recovery has been reported earlier [15,16]. After that they were mailed a 5-year questionnaire retrospectively to obtain their current health information. Of the 173 patients, 7 were excluded due to previous back surgery. The final study group followed up for five years consisted of 166 virgin lumbar disc herniation patients. The age of the patients varied from 16 to 74 years (Table 1).

The indication for the initial surgery was extensive or unbearable pain radiating down to the lower extremity or muscle weakness. In some cases also, loss of the patellar or Achilles reflex, regional sensory loss, and a positive straight leg raising test (SLR <60) were present. The diagnosis of lumbar disc herniation was based on preoperative clinical status, and spinal nerve root compression detected in magnetic resonance imaging (MRI) or computed tomography (CT) and was finally confirmed during surgery. The patients were operated on using the open mini approach described by Wood & Hanley in 1991 [17]. In the surgery, herniated fragment was extracted and thereafter, loose material from intervertebral disc space was removed.

Before surgery, the subjects completed a questionnaire including items about the duration of preoperative back and leg pain, intensity of pain (visual analog scale, VAS, scale 0–100 mm), leisure time physical activity, employment status, and physical loading at work (light, medium, heavy or very heavy work). The patients filled a questionnaire also 5 years after the surgery confirming that there were no re-operations done in other hospitals. Data on the patients' initial disc operations and re-operations during the follow-up were collected from patient files of Jyväskylä Central Hospital. In the analysis re-operations at the same side and level as the primary operation and lumbar disc operations at other sites were analyzed separately. The ethical committee of Jyväskylä Central Hospital approved the study design and all patients gave written informed consent.

### Statistics

The results were expressed as means with standard deviations (SD), and medians with interquartile ranges (IQR) or range. Statistical comparison between the groups was made by using the t-test, Mann-Whitney test (Monte Carlo p-value) and chi-Square or Fisher-Freeman-Halton test where appropriate. Kaplan-Meier estimate was used to generate the cumulative proportion of re-operation rates. 95 per cent confidence intervals of cumulative proportion was obtained by bias corrected bootstrapping (5000 replications)[18]. Cox's proportional Hazard Model with bootstrap estimate of variance was used to estimate risk for re-operation.

## Results

**Figure 1:**
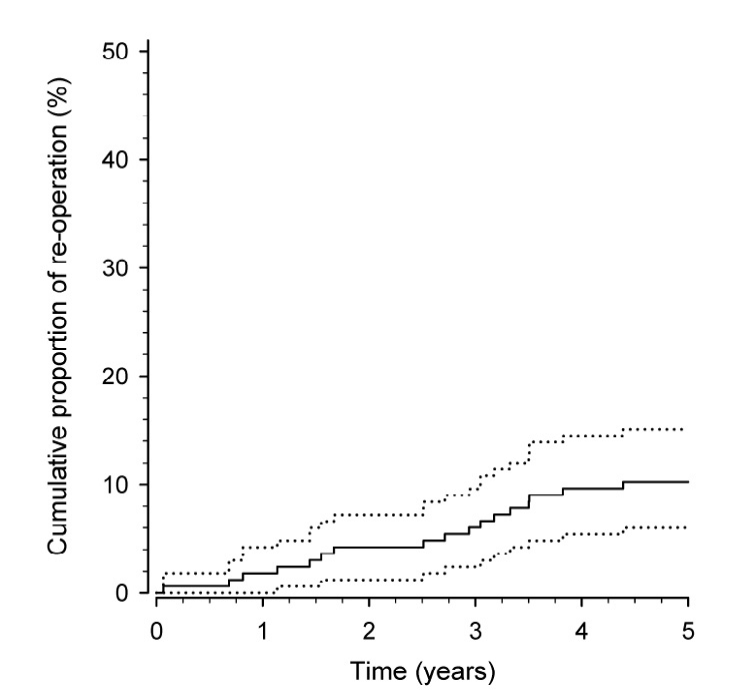
Cumulative proportion of re-operations for lumbar disc herniotion after first lumbar disc herniation surgery (dotted line shows 95% confidence interval).

**Figure 2:**
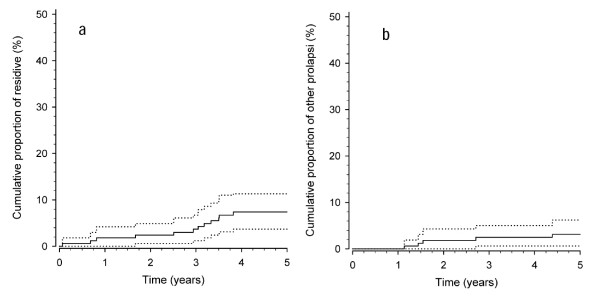
Cumulative proportion of residive prolapse occurring at the same site as the primary operation (a) and other (b) prolapses after first lumbar disc herniation surgery (dotted line shows 95% confidence interval).

Of the original 166 patients the cumulative rate of re-operations for lumbar disc herniation over the 5-year period was 10.2% (17 patients, 95% Cl 6.0 to 15.1) (Figure 1). Of those, twelve patients [7.4%; 95% Cl 3.7 to 11.3] had residive at the same side and level as the primary herniation and five [3.1 %; 95% Cl 0.6 to 6.2] had herniation at a site other than that of their primary prolapse (Figure 2). In addition to re-operated lumbar disc herniation 6 patients also underwent other back surgery during the follow-up (2 had decompressive surgery and 4 had spinal fusion). Three out of twelve residives occurred within one year, and the overall occurrence of residive lumbar disc herniations was evenly distributed over the 5 years. All primary and re-operations were done in the same hospital.

In terms of age, sex, duration of symptoms, or intensity of back and leg pain there was no statistical difference between the single-operated or re-operated patients at the time of the primary disc operation (Table 1). Similarly, no differences between the groups were found in leisure time physical activity or employment status. Of those who were employed before the first operation, 47 % in the single-operated group and 67% in the re-operated group worked in physically heavy occupations; there was no statistical difference between the groups. Also there were no differences between the single-operated and re-operated groups in the site of the disc herniation or in the proportion of patients with positive straight leg raising test before the operation (Table 2).

In the Cox proportional Hazard Model, which included age, gender, preoperative symptoms, physical activity and employment, none of the variables explained the re-operations over the five year follow-up (Table 3).

## Discussion

In the present study, the cumulative rate of re-operations for lumbar disc herniation was 10% at 5-year follow-up. Atlas et al. 2005 reported outcomes of patients with lumbar disc herniation treated surgically or nonsurgically [19]. At 10-year follow-up out of 217 surgically treated patients 25% had undergone at least one additional lumbar spine operation. Österman et al. 2003 reported an increasing cumulative risk for lumbar re-operations over time as at the one-year follow-up the risk was 7% and at the 10-year follow-up 25% [20]. A Swedish 10-year follow-up showed that 10% out of 27 576 patients underwent multiple operations for disc herniation [9]. A large Finnish study with 25 366 patients and with an average follow-up time of 4 years reported that 12% of the patients had at least one re-operation in the lumbar area [10]. In that study 76% of the first re-operations were repeated extirpations of disc herniations, 21 % decompression operations and 3% spinal fusion operations. Four percent of our patients had decompressive surgery or spinal fusion after the first lumbar disc herniation. However, the accurate comparison of the risk for re-operations between studies is not possible due to differences in samples, follow-up times and statistical methods.

In the present study the proportion of residive herniations at the same site as the primary disc herniation was 71% from all re-operated patients. This result was in line with the proportion (75%) reported by Suk et al. 2001 [14], while Silvers et al. 1994 reported the rate of residives to be 46% [21]. The relative rate of residives has varied from 1.2% to 7.4% from all lumbar reoperations [4,11,12,22,23]. However, the actual rate of residives out of all reoperations is very difficult to compare between the studies. In many of the previous studies the data collection has been made so long time ago (starting from year 1958) that the diagnostic methods, operation techniques as well as criterion for the surgery have changed during the time span. Further, the follow-up times have varied from 7 months up to 14 years and number of subjects from 28 to 1850 [4,11,12,22,23]. The present study is the only one where the subjects are followed for equal time period.

According Suk et al. 2001 the high risk of residives may be explained by the initial annular defect and the trauma to the annulus sustained during the lumbar disc surgery [14]. However, the reasons reported for residive disc herniation are conflictive. Cinotti et al. (1998) observed residive disc herniations in patients with severe disc degeneration, while recently Dora et al. (2005) reported that patients with only minor disc degeneration have a 6.8-fold increase in the risk for residive disc herniation compared to those with advanced grade IV degeneration [22,24]. In the present study the age distribution, and thus apparently also the grade of disc degeneration of all patients was wide (16–74 years) and was comparable to that of the patients with residive disc herniation (19–74 years).

Earlier studies have suggested that the origin of residive disc herniation may vary [25,26]. Early residive lumbar disc herniation is made primarily of disc material left in the intervertebral space. Gradually the disc material may also form fibrocartilaginous tissue, which can be extruded into the spinal canal. A pain-free interval of less than 12 months after the initial lumbar disc operation and slow onset of new complaints are assumed to be characteristic of an epidural fibrosis with nerve compression. In epidural fibrosis the nerve root and the dura are immobilized by the epidural scar tissue, and the pressure is exerted on the nerve root [25]. Luukkonen has also recently reported that a scar as a surgical finding was a significant factor in poor outcome in recurrent nerve compression [27]. In the present study three out of twelve residives representing possible "actual residives" occurred within one year, while the remainders occurred 2.5 years after the first lumbar disc herniation, possible reflecting the different reason for the re-operation. The distribution of levels of herniated discs in both the single-operated and re-operated patients was similar to that reported in other studies with a significant prevalence of L_4_-L_5 _and L_5_-S_1 _-levels [28,29].

In the Cox proportional Hazard Model, in which age, gender, preoperative symptoms, physical activity and employment were included, none of these variables explained the re-operations. This result is in accordance with Kara et al. (2005) with the exception that they reported lack of physical exercise to be a significant predictor of re-operation (OR 4.60; p = 0.013) [29]. However, Jansson et al. (2004) reported that patients aged 40 to 59 had an increased risk for re-operations compared with patients below age 40 or above age 60 over a median follow-up period of six years [9]. Videman and Battie (1999) concluded in their review that none of the classic occupational risk factors (heavy lifting, sitting and bending) were predictive of disc degeneration or re-operations [30]. The risk factors for primary disc herniation have been reported to be structural weakness of the annular tissue, and exposure to repetitive lifting, vibration, and smoking [31-33]. Some studies have also shown that the onset of radicular pain and recurrent herniation was related to a trauma or injury [14,2]. The limitation of the study is the low number of reoperations and thus the information of the risk factors should be considered with caution.

In conclusion, 71 percent of the re-operations for lumbar disc herniation were residive disc herniations and a minor proportion of re-operations occurred due to disc herniations at another side or level. The re-operations were not explained by age, gender, preoperative symptoms, physical activity or employment.

## Competing interests

The author(s) declare that they have no competing interests.

## Authors' contributions

AH participated in the study design, acquisition, analysis and interpretation of data and drafting of the manuscript. IK and JY participated in the study design and drafting of the manuscript. MK participated in drafting of the manuscript and HK in statistical analysis of the data and drafting of the manuscript. All authors read and approved the final manuscript.

## Pre-publication history

The pre-publication history for this paper can be accessed here:



## Figures and Tables

**Table 1:** Sociodemographic and clinical characteristics of the patients at the time of their first operation for lumbar disc herniation.

	Single operated N = 149	Re-operated N = 17	P-value
Number of males	86 (58)	8 (47)	0.40
Age, years, mean (SD)	42 (12)	41 (14)	0.70
Height, cm, mean (SD)	173 (9)	172 (8)	0.61
Body mass index, mean (SD)	26 (4)	26 (3)	0.76
Duration of back pain, months, median (IQR)	10 (3, 24)	12 (6, 117)	0.11
Duration of leg pain, months, median (IQR)	6 (3, 14)	10 (3, 15)	0.19
Intensity of pain before operation, VAS, median (IQR)			
Back pain	59 (34, 82)	76 (50, 86)	0.070
Leg pain	74 (58, 90)	76 (61, 94)	0.45
Leisure time physical activity, h/week, median (IQR)	3.5 (1.0, 6.8)	5.5 (2.9, 7.5)	0.29
Employment status, no (%):			0.48
Employed	109 (73.2)	12 (70.6)	
Unemployed	10 (6.7)	3 (17.6)	
Student	8 (5.4)	1 (5.9)	
Retired	18 (12.1)	1 (5.9)	
Other	4 (2.7)	-	

**Table 2:** The level and site of the first lumbar disc herniation operation and proportion of patients with a positive straight leg raising (SLR) test.

	Single operated N = 149	Re-operated N = 17	P-value between the groups
Level of surgery			0.74
L_1–2_	4 (2.7)	0 (0)	
L_2–3_	4 (2.7)	0 (0)	
L_3–4_	9 (6.0)	0 (0)	
L_4–5_	72 (48.3)	8 (47.1)	
L_5_-S_1_	57 (38.3)	8 (53.7)	
L_4_-L_5 _and L_5_-S_1_	3 (2.0)	1 (5.9)	
Site of first operation			0.075
right	58 (38.9)	6 (29.4)	
left	85 (57.1)	9 (53.0)	
central	7 (4.0)	2 (17.6)	
SLR before operation			0.91
right	29 (19.5)	4 (23.6)	
left	36 (24.1)	3 (17.6)	
both sides	8 (5.1)	1 (5.9)	

**Table 3:** Cox proportional Hazard Model for re-operation after first lumbar disc herniation surgery.

	Hazard Ratio	95% Confidence Interval *	p-value
Female sex	1.47	0.19 to 11.19	0.71
Age, years	0.98	0.92 to 1.04	0.53
Body mass index >27.0	1.62	0.42 to 6.29	0.36
Duration of pain, mo	1.01	0.98 to 1.02	0.66
Back pain per 10 mm on VAS	1.17	0.88 to 1.54	0.27
Leg pain per 10 mm on VAS	1.01	0.73 to 1.40	0.94
Leisure time physical activity	1.09	0.93 to 1.28	0.28
Employment	1.16	0.00 to >50	0.96
